# A cross‐sectional study of hair parameters in the occipital region of male pattern hair loss

**DOI:** 10.1111/srt.13771

**Published:** 2024-06-25

**Authors:** Yeqin Dai, Lifang Hu, Yi Wu, Tao Wang, Xiuzu Song

**Affiliations:** ^1^ School of Medicine Zhejiang University Hangzhou China; ^2^ The Department of Dermatology Hangzhou Third People's Hospital Hangzhou China; ^3^ Hangzhou Third Hospital Affiliated to Zhejiang Chinese Medical University Hangzhou China

Dear editor

Male pattern hair loss (MPHL) is characterized by progressive hair thinning in a specific pattern on the scalp in genetically predisposed individuals.^1^ The occipital part is typically not affected in androgenetic alopecia (AGA) patients, making it an ideal donor site for hair transplantation.^2^ However, there are instances where the occipital regions are also involved.^3^, ^4^ The aim of our study was to evaluate fundamental hair‐associated parameters in the occipital region of MPHL.

A total of 386 male patients diagnosed with MPHL were included in the study. All patients were categorized according to the BASP classification.^5^ The performances of C3, U1‐U3 were classified as severe (*n* = 76), M1‐2, C1, V1‐2, F1‐2 were classified as mild (*n* = 188), and the remaining cases belonged to the moderate category (*n* = 122).We selected four points representing the occipital region, including the occipital protuberance, 2 cm to the left, right, and below it (Figure 1). The analyzed factors included hair density, the number of vellus‐like hairs, and the ratio of single to compound hairs. Hair shafts with a diameter <30 µm were classified as vellus‐like hairs.^3^ Hair density was calculated after removing vellus‐like hairs.

**FIGURE 1:**
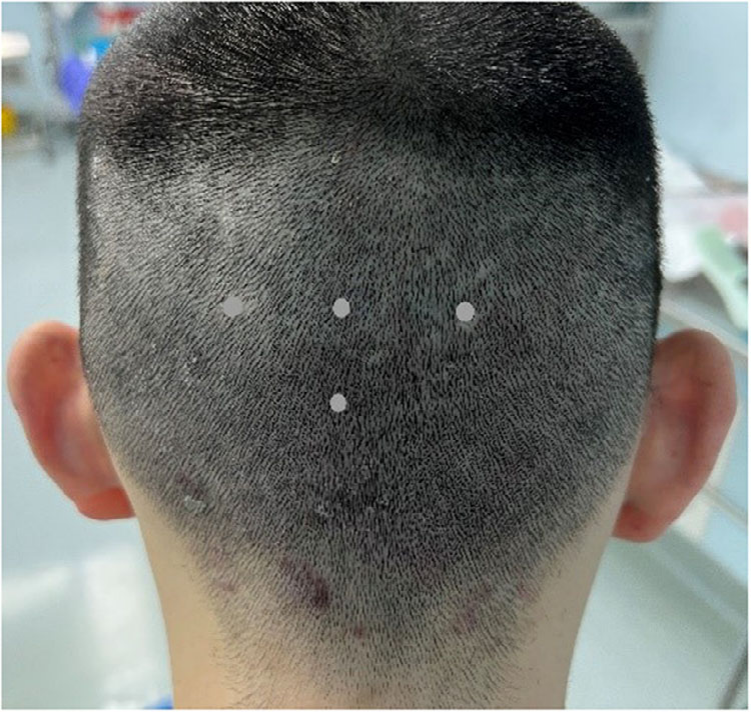
Four points representing the occipital region, including the occipital protuberance, 2 cm to the left, 2 cm to the right, and 2 cm below it.

There were statistically significant differences in mean ages between the mild and the moderate (*p* = 0.000), the mild and the severe (*p* = 0.000), but no statistically significant difference between the moderate and the severe (*p* = 0.17).

Compared among the three groups, there were statistically significant differences in Body Mass Index (BMI) (*p* = 0.004). The differences were between the mild and the severe (*p* = 0.002), with no statistically significant differences within the mild and the moderate (*p* = 0.08), and the moderate and the severe (*p* = 0.065).

The mean hair density at the occipital area is shown in the Table 1. The mean hair density at the occipital area of the severe group is lower than the mild (*p* = 0.049) and the moderate (*p* = 0.014). No statistically significant difference between the mild and the moderate. There were no statistically significant differences in the single to compound hairs ratio between the groups.

**TABLE 1:** Variables and hair counts in the occipital scalp of patient subgroups with varying severity of androgenetic alopecia.

	Number of cases	Age, y, mean (SD)	BMI(Kg/m^2^)	Hair density(*n*/cm^2^) (vellus hair‐like hair excluded)	Single to compound hairs ratio
Mild	188	28.79 ± 5.22	23.43 ± 3.74	119.08 ± 22.19	0.22 ± 0.13
Moderate	122	32.28 ± 6.65	24.05 ± 2.84	121.82 ± 22.23	0.24 ± 0.11
Severe	76	34.63 ± 6.77	25.24 ± 3.38	110.93 ± 18.86	0.25 ± 0.13

Our study found that patients with mild MPHL were younger, indicating that young people may pay more attention to their hair and appearances. Although there was a difference in age between the mild and the moderate, there was no difference in occipital hair density between the two groups. There was no age difference between the moderate and the severe; however, there was a difference in hair density between the two groups, suggesting that severe patients had lower occipital hair density. The difference in BMI between the mild and the severe groups may be related to age, as younger people may be slimmer. There is a decrease in hair density in the occipital region as MPHL progresses, possibly associated with an increase in vellus‐like hairs, as hair density in our study includes the removal of vellus‐like hairs. We also recommend that hair transplants be performed early to ensure the quality of the donor zone.

## CONFLICT OF INTEREST STATEMENT

The authors declare no conflicts of interest.

## PATIENT CONSENT STATEMENT

Informed patient consent was obtained for the publication of all patient photographs and medical information at the time of article submission.

## Data Availability

Data sharing is not applicable to this article as no new data were created or analyzed in this study.
